# Downregulation of *OPCML* is associated with activation of AKT signaling and aggressive phenotypes in glioblastoma cells

**DOI:** 10.3389/fonc.2025.1710073

**Published:** 2026-01-05

**Authors:** Zhixin Liu, Chunhua Xu, Wu Zhou, Bilin Lin, Yihao Liu, Wenrui Wu

**Affiliations:** 1 Nanchang County People’s Hospital, Nanchang, Jiangxi, China; 2 Department of Neurosurgery, The First Affiliated Hospital, Fujian Medical University, Fuzhou, Fujian, China; 3 Department of Neurosurgery, The First Affiliated Hospital, Jiangxi Medical College, Nanchang University, Nanchang, Jiangxi, China; 4 Department of Rehabilitation, Jiangxi Provincial Hospital of Traditional Chinese Medicine, Nanchang, Jiangxi, China; 5 The Second Affiliated Hospital, Jiangxi Medical College, Nanchang University, Nanchang, Jiangxi, China; 6 Department of Neurosurgery, Xiangyang Central Hospital, Affiliated Hospital of Hubei University of Arts and Science, Xiangyang, China

**Keywords:** AKT–mTOR, glioblastoma, immune infiltration, *OPCML*, single-cell RNA-seq

## Abstract

**Background:**

*OPCML* (opioid-binding protein/cell adhesion molecule-like), a glycosylphosphatidylinositol (GPI)-anchored IgLON adhesion molecule with brain-enriched expression, has tumor-suppressive roles in several epithelial cancers; however, its role in glioblastoma (GBM) biology is unclear.

**Methods:**

We integrated two bulk microarray cohorts to derive a reproducible GBM signature and reanalyzed single-cell RNA sequencing (scRNA-seq) data to localize *OPCML* at single-cell resolution. The tissue distribution and clinical associations were evaluated using the Human Protein Atlas (HPA) and The Cancer Genome Atlas (TCGA), with survival modeling and a nomogram. The co-expression, STRING-based protein interaction, and Gene Ontology (GO)/Kyoto Encyclopedia of Genes and Genomes (KEGG) enrichment analyses outlined the molecular context. Immune infiltration was profiled using single-sample gene set enrichment analysis (ssGSEA) and cross-checked on TIMER2. Functional validation used a single *OPCML* small interfering RNA (siRNA) with a non-targeting siRNA control (siNC) in U87 and U251 cells with Transwell invasion, wound healing, colony formation, CCK-8 proliferation, and Western blotting for p-AKT and p-mTOR, with LY294002 rescue.

**Results:**

*OPCML* was consistently downregulated in GBM and across multiple cancers. Within GBM, a lower expression was associated with higher grade, older age, isocitrate dehydrogenase (*IDH*) wild type, and poorer overall survival. At the single-cell level, the *OPCML* transcripts were largely confined to a neuron–glia hybrid population and were scarce in classical malignant clusters. Genome-wide correlations in GBM showed positive links to extracellular matrix/synaptic programs and negative links to cell cycle/DNA replication pathways. *In vitro*, *OPCML* knockdown increased the invasion, migration, clonogenic growth, and CCK-8 readouts and was associated with elevated p-AKT and p-mTOR. PI3K inhibition reversed the signaling changes. Pan-cancer, *OPCML* tracked with denser immune signatures, whereas in GBM it was inversely correlated with multiple effector populations and with cancer-associated fibroblast (CAF) estimates across deconvolution methods.

**Conclusions:**

*OPCML* marks a neuron-leaning, less aggressive state and is associated with the regulation of PI3K–AKT–mTOR signaling in GBM. Loss of *OPCML* aligns with proliferative programs and a GBM-specific immune pattern. These data nominate *OPCML* as a prognostic marker and a surface-level modulator that could be leveraged alongside RTK/PI3K axis inhibitors in GBM.

## 1 Introduction

Glioblastoma (GBM) is the most aggressive primary brain tumor in adults. Standard chemoradiotherapy after maximal safe resection gives a median overall survival of roughly 14–16 months, and durable control is uncommon (1, 2). Large-scale profiling by The Cancer Genome Atlas (TCGA) and subsequent cohorts showed that recurrent lesions converge on p53, RB, and RTK/PI3K signaling, providing a genomic basis for pathway co-activation and therapeutic resistance (3, 4). At the cellular level, malignant cells cycle among a limited set of transcriptional states under genetic and microenvironmental pressure, and the brain tumor immune milieu is dominated by myeloid populations with restricted effective T-cell activity. Together, these features argue for targets that sit at the interface of receptor signaling, adhesion programs, and the local microenvironment (3, 5).

*OPCML* (opioid-binding protein/cell adhesion molecule-like) is a glycosylphosphatidylinositol (GPI)-anchored IgLON family member with strong brain expression and established roles in neural adhesion and synaptic organization (6). Beyond physiology, *OPCML* shows tumor-suppressive activity in epithelial cancers. It is frequently silenced by promoter hypermethylation, which was first described in ovarian cancer and subsequently across multiple carcinomas (7, 8). Mechanistically, *OPCML* acts as a cell surface “repressor–adaptor” for selected receptor tyrosine kinases (RTKs), promoting their endocytic removal and dampening downstream signaling (9). In preclinical models, *OPCML* can potentiate the responses to anti-EGFR/anti-HER2 therapy in HER2-positive ovarian and breast cancer and, together with the phosphatase PTPRG (protein tyrosine phosphatase receptor type G), helps inactivate AXL-dependent signaling—findings that speak to both biology and translational relevance (10, 11). These observations position *OPCML* at the interface of receptor signaling and adhesion programs, where GBM pathobiology is most active. Notably, similar links between *OPCML* and RTK–PI3K/AKT signaling have been described outside the nervous system. In epithelial models, *OPCML* restrains surface RTKs (e.g., HER2/EGFR family members and AXL) and dampens the downstream PI3K–AKT outputs. Restoring *OPCML* can also sensitize tumors to anti-EGFR/anti-HER2 therapy (9). Together with reports of the PTPRG-dependent inactivation of AXL in *OPCML*-positive cells, these data offer a mechanistic backdrop for asking whether a comparable axis operates in GBM.

Here, we position *OPCML* within the GBM landscape by combining independent bulk cohorts with single-cell RNA sequencing (scRNA-seq), then examining the consequences of *OPCML* loss in GBM cell lines. We examine expression across normal brain and cancers, evaluate survival associations, map the co-expression and interaction contexts, and ask whether loss of *OPCML* promotes aggressive phenotypes by releasing PI3K–AKT–mTOR signaling. As the immune context is pivotal in GBM, we also relate the *OPCML* levels to immune cell infiltration patterns using established deconvolution frameworks.

## 2 Materials and methods

### 2.1 Study design and datasets

We combined public transcriptomics with *in vitro* assays. The bulk microarrays GSE16011 and GSE116520 were analyzed for differential expression, and scRNA-seq GSE84495 was reprocessed to place *OPCML* at cellular resolution. Normal tissue expression came from the HPA and pan-cancer/GBM cohorts from TCGA. Bioinformatics results were followed by *OPCML* knockdown in U87 and U251 cells to assess the proliferation, migration/invasion, clonogenicity, and PI3K–AKT signaling. All statistics used R 4.3.2.

### 2.2 Bulk and single-cell transcriptomics

The expression matrices and annotations were downloaded from the Gene Expression Omnibus (GEO). When CEL files were available, the data were Robust Multi-array Average (RMA)-normalized and log_2_-transformed; otherwise, GEO-normalized matrices were used. Probes were mapped to gene symbols (the highest mean probe was retained). Differential expression (GBM *vs*. non-tumor brain) used limma with *p* < 0.05 and |log_2_FC| ≥ 2. For scRNA-seq, the counts were processed in Seurat with explicit quality control (QC) thresholds (cells were retained if the nFeature_RNA was 200–6,000, the nCount_RNA was 500–50,000, and the percent.mt was <10%; genes detected in less than three cells were excluded), log-normalized, and reduced using the top 2,000 highly variable genes (HVGs) for principal component analysis (PCA). Graph-based clustering and uniform manifold approximation and projection (UMAP) were used for visualization and annotation with canonical markers. The distribution of *OPCML* was displayed using feature/violin plots. Heat maps and other figures were generated with ggplot2/pheatmap.

### 2.3 Expression, survival, and pathway context

HPA normalized transcripts per million (nTPM) values summarized the tissue distribution of *OPCML*. In TCGA, tumor–normal and multigroup comparisons used the Wilcoxon and Kruskal–Wallis tests with Dunn’s *post-hoc* test, where needed. Multiple testing used Benjamini–Hochberg. Survival was assessed using the Kaplan–Meier (log-rank) and Cox models [age, sex, and isocitrate dehydrogenase (*IDH*), when available]. Pan-cancer Spearman’s co-expression was computed in R [Benjamini–Hochberg false discovery rate (BH-FDR) control]. Protein interactions were queried directly on STRING and exported as screenshots. For Gene Ontology (GO)/Kyoto Encyclopedia of Genes and Genomes (KEGG) enrichment, clusterProfiler was used with FDR < 0.05 (Benjamini–Hochberg), as well as the universe = detected genes after low-expression filtering. The parameters minGSSize = 10 and maxGSSize = 5,000 were applied.

### 2.4 Survival analysis and nomogram

Patients were split at the median into the high-*OPCML* and low-*OPCML* groups. Overall survival was analyzed using Kaplan–Meier with log-rank testing (survival and survminer). Univariable and multivariable Cox models included the available clinical covariates (e.g., age, sex, and *IDH* status). A prognostic nomogram was built with rms, with internal bootstrap calibration (*B* = 1,000) at 6, 12, and 24 months. The calibration plots showed agreement between the model-predicted survival probabilities and the Kaplan–Meier estimates calculated within quintiles of the linear predictor (risk groups).

### 2.5 Immune infiltration

The immune cell scores were computed using single-sample gene set enrichment analysis (ssGSEA) (gene set variation analysis, GSVA). Deconvolution on TIMER2 (e.g., EPIC, MCP-counter, quanTIseq, and xCell) was performed to cross-check the immune and stromal estimates, including the cancer-associated fibroblasts (CAFs). Associations with *OPCML* used Spearman’s tests and the high/low group comparisons (Wilcoxon) with BH-FDR correction.

### 2.6 Cell culture and reagents

U87-MG, U251, and SVG p12 were maintained in Dulbecco’s modified Eagle’s medium (DMEM) + 10% fetal bovine serum (FBS) + 1% penicillin/streptomycin at 37°C and 5% CO_2_. For signaling assays, the cells were serum-starved for 4 h and then restimulated with epidermal growth factor (EGF) 20 ng/ml for 10 min. LY294002 (10 µM) was added 1 h before stimulation, where indicated (vehicle: dimethyl sulfoxide, DMSO). Unless stated, the materials were sourced as follows: DMEM, FBS, 4% paraformaldehyde, and crystal violet (Solarbio, Beijing, China); *OPCML* siRNA and quantitative PCR (qPCR) primers (Tsingke Biotech, Beijing, China); primary antibodies p-AKT (Ser473), p-mTOR (Ser2448), OPCML, and β-actin (Wuhan Sanying Biotech, Wuhan, China); Transwell inserts (8 µm), 24-well plates, Matrigel (Corning, New York, NY, USA); qPCR reagents (Yeasen Biotechnology, Shanghai, China); and CCK-8 kit (Shanghai Sheng'er Biotechnology Co., Ltd., Shanghai, China). All other consumables (e.g., tubes and dishes) were from Wuhan Sevier Biotechnology (Wuhan, China).

### 2.7 siRNA transfection

A single *OPCML* siRNA (H4978-siOPCML-1; sense 5'-GUAUGACGAAGGUCCGUAC-3' dTdT; antisense 5'-GUACGGACCUUCGUCAUAC-3' dTdT) and a non-targeting siRNA control (siNC; sense 5'-UUCUCCGAACGUGUCACGU-3' dTdT; antisense 5'-ACGUGACACGUUCGGAGAA-3' dTdT) were transfected with Lipofectamine RNAiMAX at a concentration of 10 nM siRNA and 5 µl Lipofectamine RNAiMAX per well at ~50%–60% confluence. The assays were performed 48 h post-transfection. For the inhibitor experiments, the groups were siNC + DMSO, siOPCML + DMSO, siNC + LY294002 (10 µM, 1 h), and siOPCML + LY294002, with vehicle (DMSO) matched across siRNA conditions.

### 2.8 qRT-PCR

Total RNA was isolated with TRIzol, and 1 µg was reverse-transcribed using random primers. qPCR was run with SYBR Green (Yeasen, Shanghai, China) under the manufacturer’s cycling conditions. *β-actin* was used as the reference gene, and relative expression was calculated with the 2^−ΔΔ^*^C^*^t^ method. The *OPCML* primers (synthesized by Beijing Tsingke Biotech, Beijing, China) were: forward 5'-GACCCTGCTGTGTCTTGCTA-3' and reverse 5'-CGGACTGGTCTCGCTTGATG-3' (134-bp amplicon). All siRNAs carried 3' dTdT overhangs.

### 2.9 Western blot

Cells were lysed in RIPA (protease/phosphatase inhibitors). Approximately 40 µg protein per lane was separated by SDS-PAGE, transferred into PVDF, blocked (5% bovine serum albumin, BSA), and probed with p-AKT, p-mTOR, OPCML, and β-actin (Wuhan Sanying, Wuhan, China), followed by horseradish peroxidase (HRP) secondary antibody and enhanced chemiluminescence (ECL) detection. The band intensities were quantified in ImageJ and normalized to that of β-actin.

### 2.10 Transwell invasion assay

Corning 24-well chambers (8 µm) were Matrigel-coated. At 48 h post-transfection, 5 × 10^4^ cells in serum-free medium were seeded (upper), with 10% FBS medium used as a chemoattractant (lower). After 24 h, the non-invading cells were removed and the membranes were fixed (4% paraformaldehyde), stained (crystal violet), and the invaded cells counted in five fields/insert.

### 2.11 Wound healing migration assay

Confluent monolayers in six-well plates were scratched with a 200-µl pipette tip and washed with phosphate-buffered saline (PBS). The cells were maintained in serum-free medium for 24 h to promote wound closure. Images were taken at 0 and 24 h at marked positions, and wound closure (percentage) was calculated relative to the baseline area using ImageJ software by measuring the difference in area between time points.

### 2.12 Colony formation assay

After treatments, 500 cells were seeded per well in six-well plates and cultured for 12 days, with medium changes every 3 days. Colonies were fixed and stained with crystal violet. Clusters containing ≥50 cells were considered as colonies and counted manually under a microscope. The number of colonies was normalized to the control condition.

### 2.13 Cell proliferation (CCK-8)

The cells were seeded in 96-well plates (~2 × 10^3^ cells/well). CCK-8 (WST-8) reagent (Shanghai Shenger Technology, Shanghai, China) was added according to the manufacturer’s protocol, and absorbance at 450 nm was read at 2 and 4 h. The CCK-8 readout reflects the mitochondrial dehydrogenase-dependent reduction of WST-8 and therefore measures the metabolic activity of viable cells. In this study, it was used as an indirect indicator of proliferation/viable cell number. As the per-cell metabolic activity can change independently of cell number (e.g., after pathway perturbations), we report and interpret the CCK-8 data alongside clonogenic growth and migration/invasion assays and treat the signal primarily as a metabolic proxy.

### 2.14 PI3K–AKT pathway measurements

To accentuate signaling differences, pathway readouts followed the serum starvation (4 h) → EGF restimulation (20 ng/ml, 10 min) workflow. In LY294002 experiments (10 µM, 1 h pretreatment), control cells received DMSO. The Western blots for p-AKT and p-mTOR were quantified and normalized to β-actin.

### 2.15 Statistics and software

Comparisons of two groups used Student’s *t*-test or Wilcoxon, as appropriate, while multigroup comparisons used one-way ANOVA + Tukey or Kruskal–Wallis + Dunn’s. Correlations used Spearman’s *ρ*. Multiple testing was controlled using Benjamini–Hochberg. Analyses/plots were performed in R 4.3.2 with limma, Seurat, GSVA, clusterProfiler, survival, survminer, rms, ggplot2, and pheatmap. Significance was set at *p* < 0.05 or FDR < 0.05.

## 3 Results

### 3.1 Integrated bulk and single-cell transcriptomic analyses nominate *OPCML* as a robust GBM-related gene

**Figure 1:**
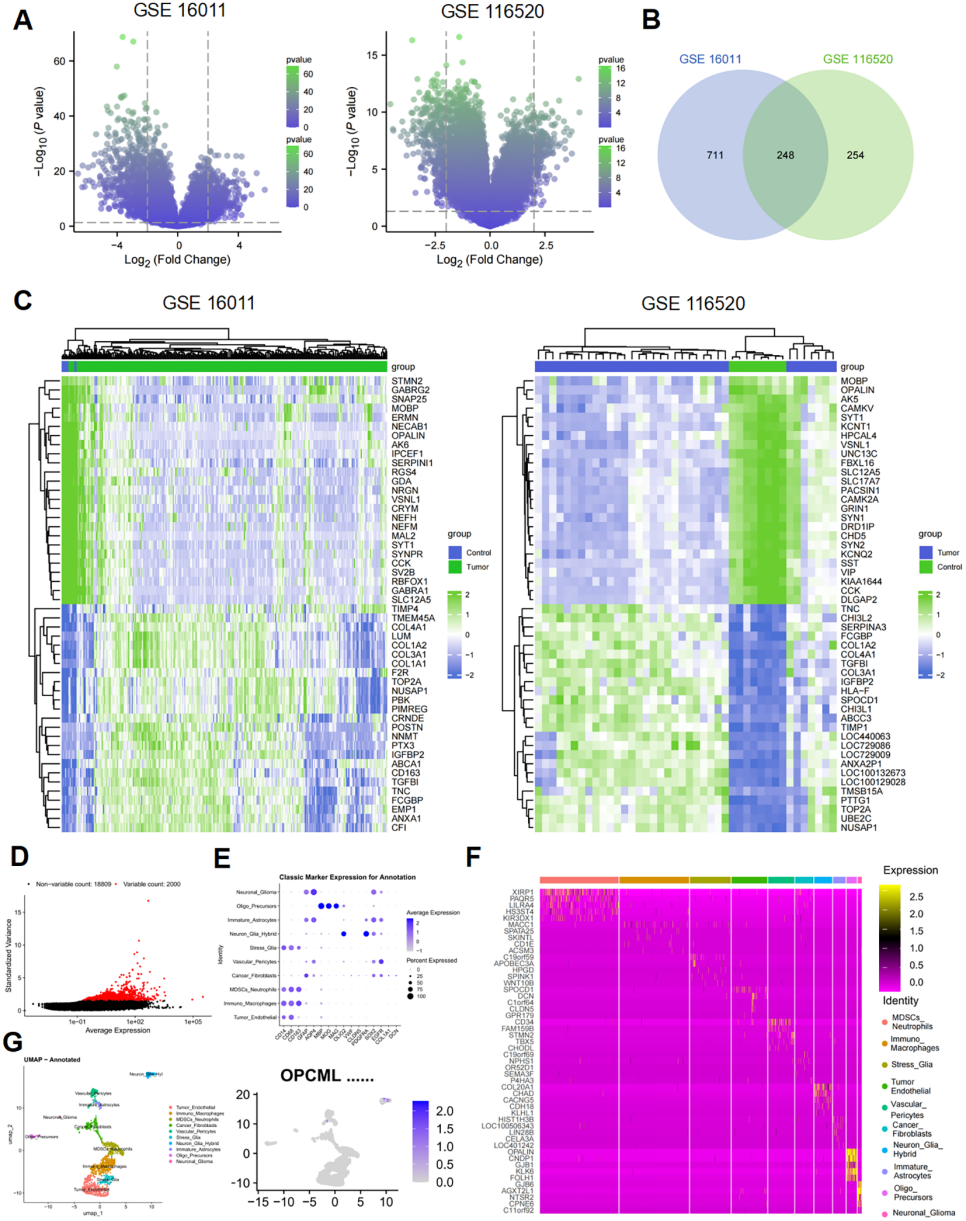
Integrated bulk and single-cell analyses nominate *OPCML* as a glioblastoma (GBM)-related gene. **(A)** Volcano plots for GSE16011 and GSE116520 (limma; with cutoffs *p* < 0.05 and |log_2_FC| ≥ 2). **(B)** Venn diagram showing 248 overlapping differentially expressed genes (DEGs) defining a core GBM signature. **(C)** Heat maps of the top 25 dysregulated genes per dataset (*z*-score, Euclidean distance, and complete linkage). **(D)** Selection of 2,000 highly variable genes (HVGs) in GSE84495 after standard quality control (QC). **(E)** Single-cell clustering and annotation by canonical markers into 10 major populations. **(F)***Z*-score heat map across annotated clusters. **(G)** Uniform manifold approximation and projection (UMAP) feature map showing *OPCML* transcripts largely confined to a small neuron–glia hybrid population and scarce in classical malignant clusters (e.g., stress–glia and oligo-precursor).

Two independent microarray cohorts were analyzed to establish a reproducible GBM transcriptional signature. Using *p* < 0.05 and |log_2_FC| ≥2 as cutoffs, 959 and 502 differentially expressed genes (DEGs) were obtained, respectively. Their distribution is shown in the volcano plots (**Figure 1A**). The overlap between the two DEG sets comprised 248 genes (**Figure 1B**), defining a robust core signature independent of platform or cohort. Unsupervised hierarchical clustering of the 25 most dysregulated genes in each dataset clearly separated the tumor from the control samples (**Figure 1C**). To contextualize this finding at single-cell resolution, the RNA-seq dataset GSE84495 was reexamined after QC. A total of 2,000 HVGs were selected (**Figure 1D**), and 10 major cell clusters were annotated with canonical markers (**Figure 1E**) and displayed in a *z*-score heatmap (**Figure 1F**). UMAP revealed that the *OPCML* transcripts were virtually absent from classical malignant clusters such as Stress_Glia and Oligo_Precursors. Instead, expression was confined to a small Neuron_Glia_Hybrid population (**Figure 1G**). This restricted distribution reconciles the bulk-level downregulation with intratumoral heterogeneity and implies that *OPCML* may characterize a neuron-like transitional state with potential tumor-suppressive properties.

### 3.2 Tissue distribution and clinicopathological correlates of *OPCML* expression

**Figure 2:**
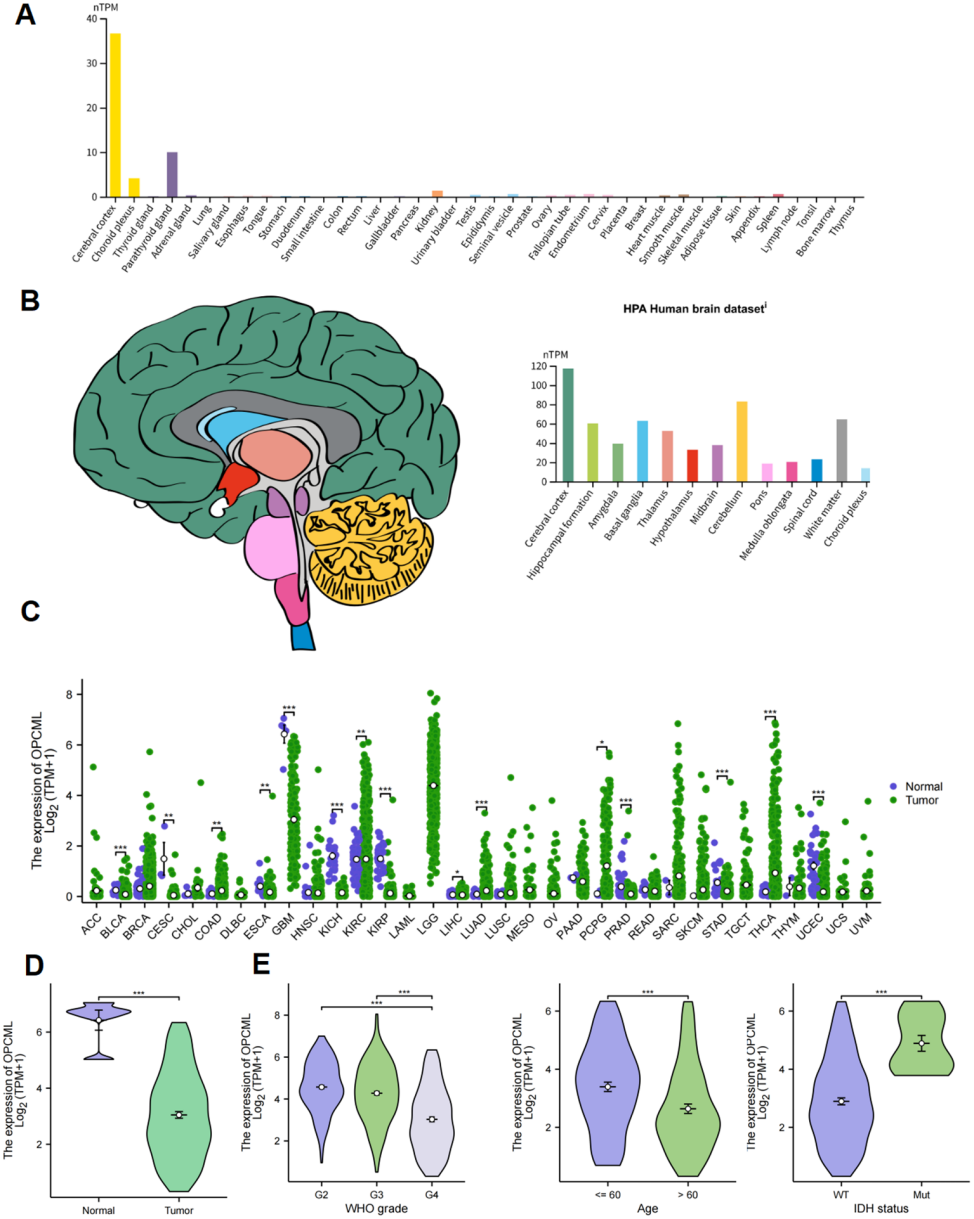
Tissue distribution and clinicopathological correlates of *OPCML*. **(A)** Normal organ expression from the Human Protein Atlas (normalized transcripts per million, nTPM), with the cerebral cortex showing the highest level. **(B)** Regional brain expression highlighting the cortex, hippocampal formation, and cerebellum. **(C)** The Cancer Genome Atlas (TCGA) pan-cancer comparison of tumor *vs*. matched normal (Wilcoxon rank-sum), with the majority of cancers showing a reduced *OPCML*. **(D)** TCGA glioblastoma (GBM) *vs*. non-neoplastic brain (Wilcoxon). **(E)** GBM stratifications: WHO grade (II–IV), age (<60 *vs*. ≥60 years), and *IDH* status (mutant *vs*. wild type) (Kruskal–Wallis with Dunn’s *post-hoc*, where applicable). *Boxes* show the median and interquartile range (IQR) and *whiskers* 1.5× IQR. *P*-values are Benjamini–Hochberg adjusted, where indicated. The asterisk * represents p<0.05, the double asterisk ** represents p<0.001, and the triple asterisk *** represents p<0.0001.

The HPA samples showed that *OPCML* expression is largely restricted to neural tissue. Among 37 normal organs, the cerebral cortex displayed the highest level (~37 nTPM) (**Figure 2A**), and within the brain the cortex, hippocampal formation and the cerebellum were the most enriched regions (**Figure 2B**). The log_2_-transformed TPM data from 33 TCGA cancer types revealed, by Wilcoxon rank-sum testing, a significant reduction of *OPCML* transcripts in the majority of tumors compared with their matched normal tissues (**Figure 2C**), with the sharpest declines in cholangiocarcinoma, esophageal carcinoma, and GBM (all *p* < 0.001). In the TCGA-GBM cohort, the tumor samples likewise showed lower expression than the non-neoplastic brain (*p* < 0.001) (**Figure 2D**). Stratification by WHO grade, patient age, and *IDH* mutation status indicated a stepwise decrease from grade II to grade IV, a diminished expression in patients older than 60 years, and a marked loss in *IDH*-wild-type tumors (all *p* < 0.001) (**Figure 2E**).

### 3.3 Prognostic relevance and molecular network of *OPCML*

**Figure 3:**
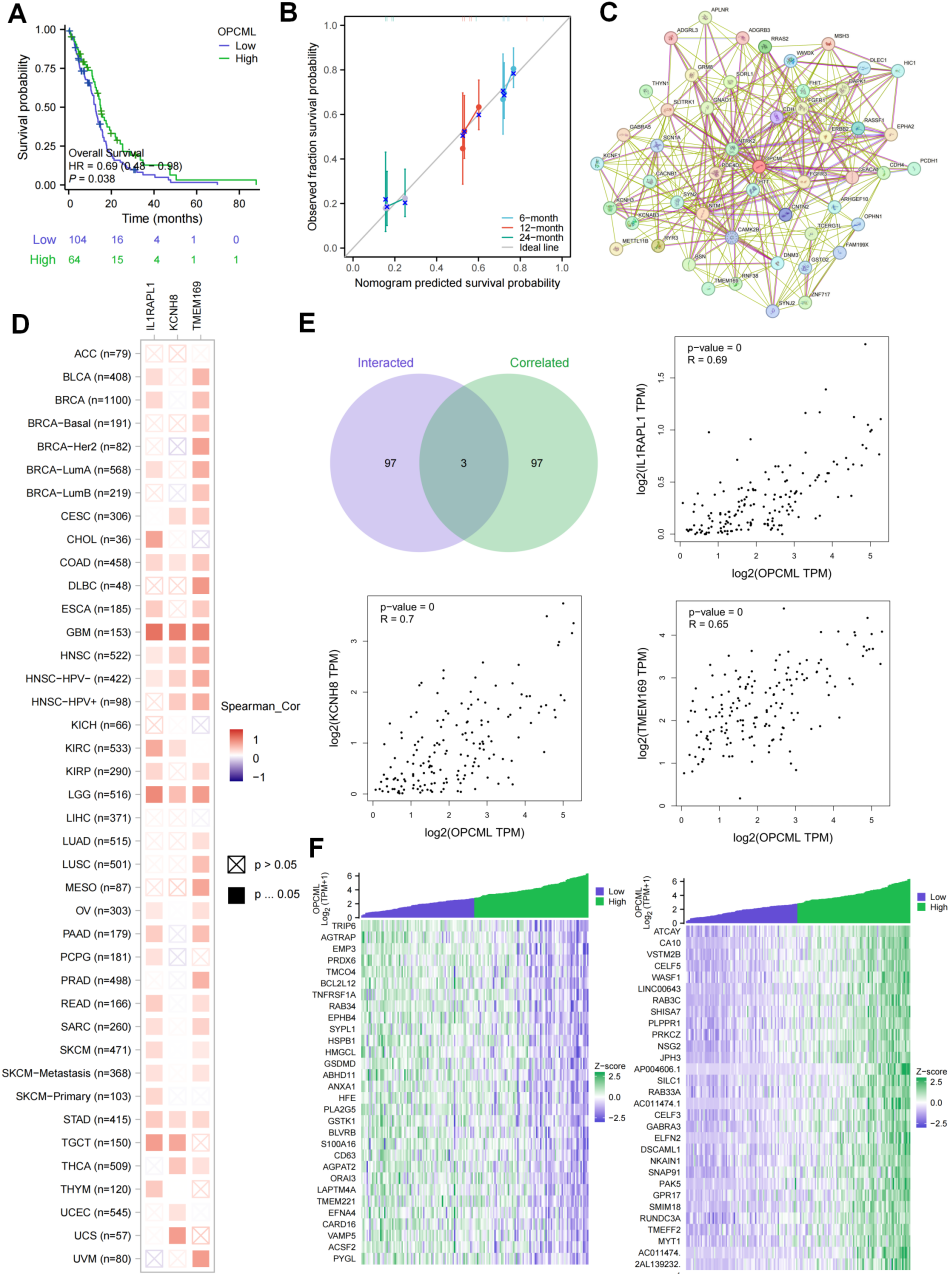
Prognostic relevance and molecular context of *OPCML*. **(A)** Kaplan–Meier curves for overall survival in The Cancer Genome Atlas (TCGA) glioblastoma (GBM) (median split, log-rank test). **(B)** Multivariable Cox model and nomogram with bootstrap calibration at 6, 12, and 24 months. **(C)** STRING protein–protein interaction map placing *OPCML* within an adhesion/synapse-related cluster (exported directly from STRING). **(D)** Pan-cancer co-expression analysis identifying *IL1RAPL1*, *KCNK9*, and *TMEM169* as transcripts consistently tracking with *OPCML*. **(E)** Scatter plots in GBM confirming positive *OPCML*–partner correlations (Spearman’s *ρ*). **(F)** Gene set enrichment of the *OPCML*-correlated genes in GBM: positive correlations enriched for extracellular matrix (ECM) organization and synaptic signaling and negative correlations enriched for cell cycle and DNA replication pathways [Gene Ontology/Kyoto Encyclopedia of Genes and Genomes (GO/KEGG), false discovery rate (FDR) < 0.05].

A high *OPCML* expression was linked to longer overall survival in TCGA glioblastoma, and multivariable Cox modeling confirmed the gene as an independent protective factor. A nomogram that combined *OPCML* with standard clinical variables showed close agreement between the predicted and the observed survival at 6, 12, and 24 months (**Figures 3A, B**). To explore its molecular context, we generated a protein–protein interaction network with STRING, which revealed *OPCML* at the center of an adhesion- and synapse-related cluster (**Figure 3C**). Across 33 TCGA tumor types, the pan-cancer co-expression analysis identified three transcripts—*IL1RAPL1*, *KCNK9*, and *TMEM169*—that consistently tracked with *OPCML* (**Figure 3D**). The same trio emerged at the intersection of the co-expression and interaction datasets, with the GBM scatter plots confirming strong positive correlations (all *p* < 0.001) (**Figure 3E**). Finally, a genome-wide correlation scan in GBM showed that the genes positively associated with *OPCML* are enriched for extracellular matrix (ECM) organization and synaptic signaling, whereas the negatively associated genes cluster in cell cycle and DNA replication pathways (**Figure 3F**), suggesting that a reduced *OPCML* expression accompanies proliferative programs in GBM.

### 3.4 *OPCML* knockdown promotes the proliferation and invasion of glioblastoma cells

**Figure 4:**
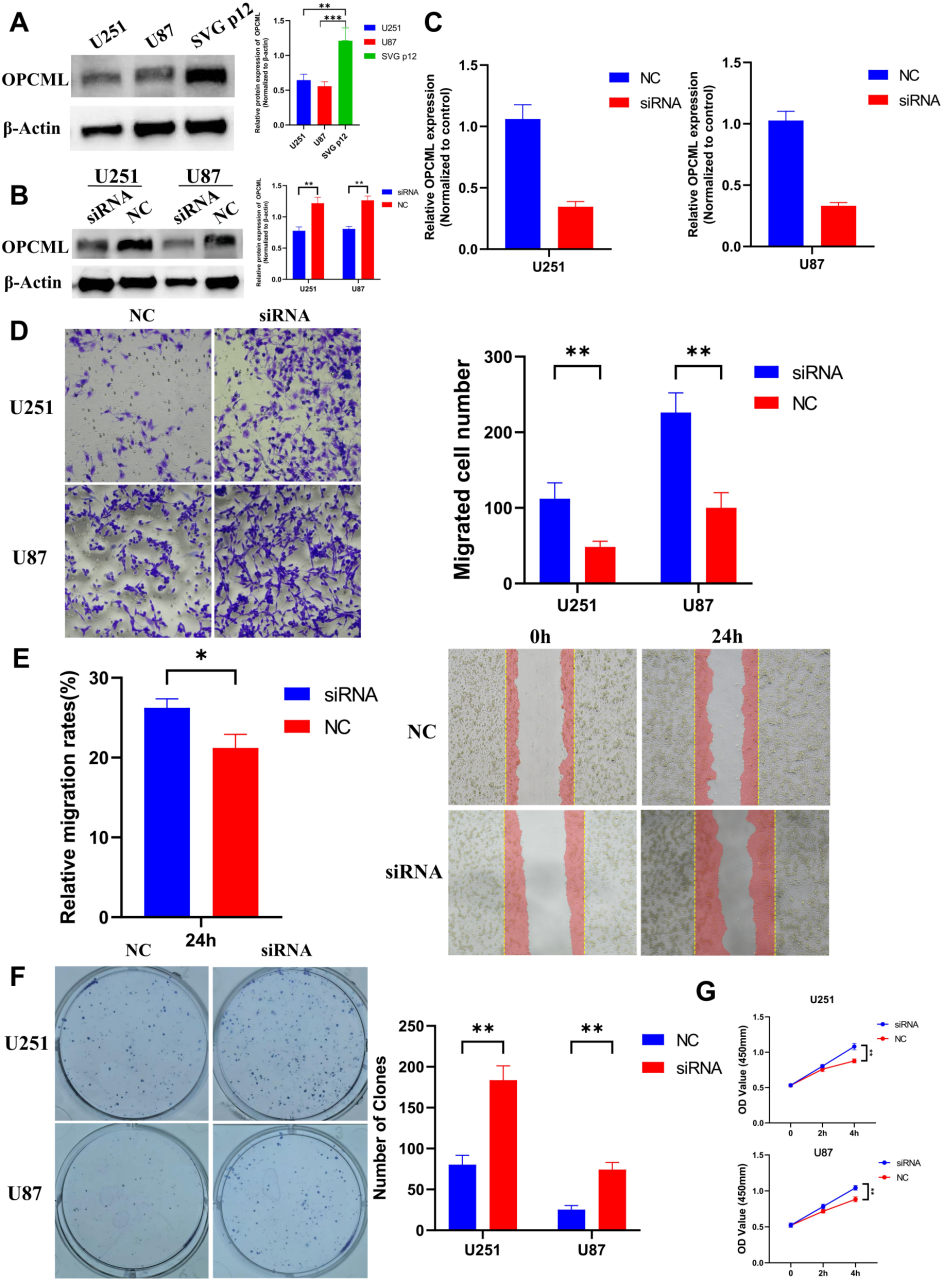
*OPCML* knockdown enhances the invasion, migration, and growth of glioblastoma (GBM) cells. **(A)** Baseline *OPCML* protein in U87, U251, and normal glial SVG p12, with the GBM lines showing lower levels. **(B, C)** Validation of *OPCML* knockdown in U87 and U251 by quantitative PCR (qPCR) and Western blot (*β-actin* loading control). **(D)** Transwell invasion increases after *OPCML* silencing. **(E)** Wound healing assays showing faster closure with *OPCML* knockdown. **(F)** Colony formation increases in knockdown cells (colonies ≥50 cells). **(G)** CCK-8 readouts (OD_450_) at 2 and 4 h are higher after *OPCML* knockdown. Data are the mean ± SEM. Statistics as indicated in panels (two-tailed tests). The asterisk * represents p<0.05 and the double asterisk ** represents p<0.001.

We first profiled the baseline OPCML protein in U251, U87, and SVG p12. U251/U87 showed lower levels than SVG p12 (**Figure 4A**). *OPCML* was silenced using a single siRNA, with siNC as the control. qPCR and immunoblotting confirmed efficient knockdown (**Figures 4B, C**). Relative to siNC, loss of *OPCML* increased Transwell invasion (**Figure 4D**), accelerated wound closure (**Figure 4E**), and raised the colony counts (**Figure 4F**). The CCK-8 OD_450_ values at 2 and 4 h were also higher in siOPCML than in siNC in both lines (**Figure 4G**).

### 3.5 *OPCML* restrains GBM progression by limiting AKT signaling

**Figure 5:**
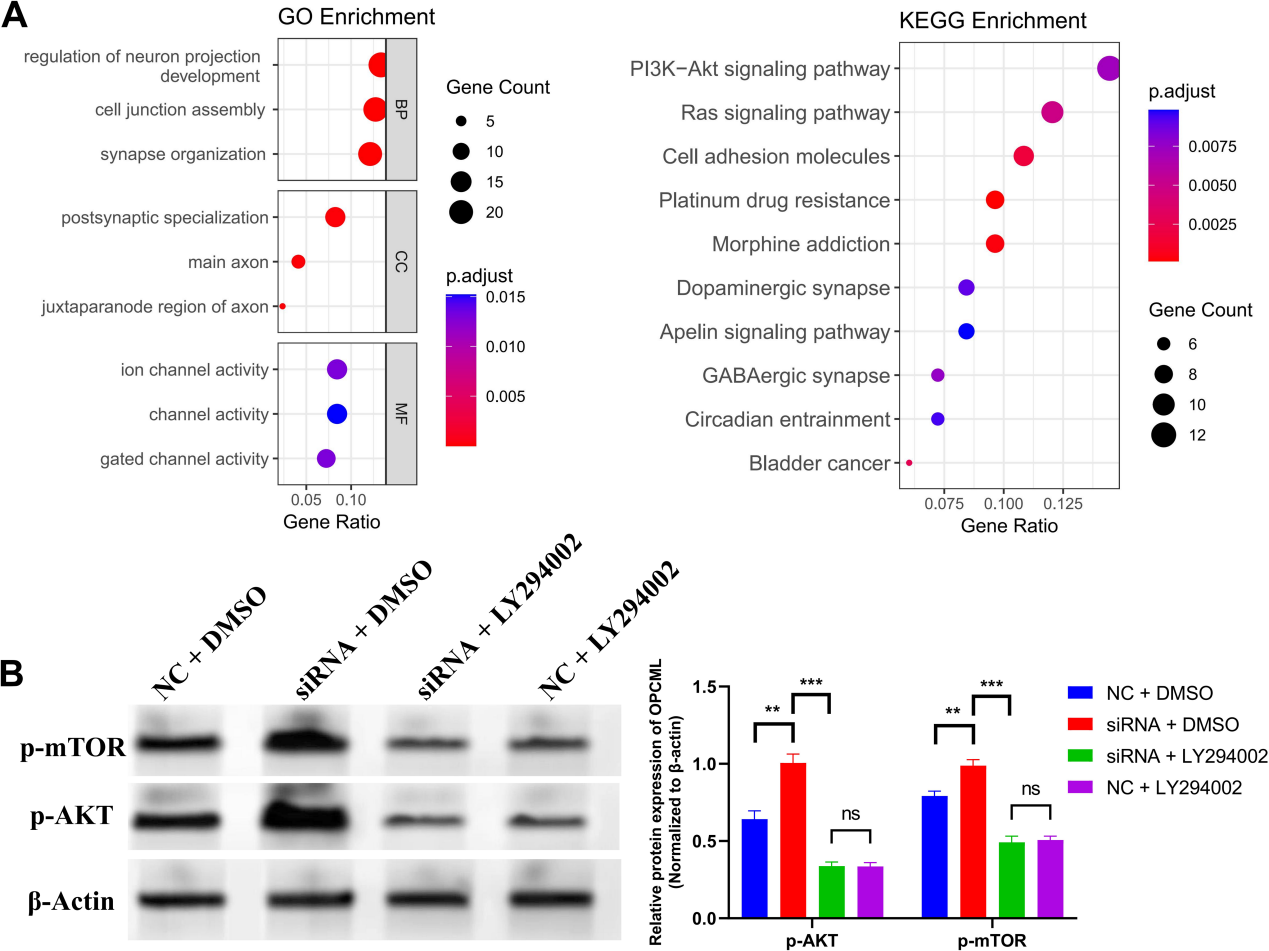
*OPCML* restrains the PI3K–AKT pathway in glioblastoma (GBM) cells. **(A)** Gene Ontology (GO)/Kyoto Encyclopedia of Genes and Genomes (KEGG) enrichment based on two correlation-derived gene sets: The Cancer Genome Atlas (TCGA) pan-cancer *OPCML* co-expression list and the TCGA-GBM OPCML co-expression list (each, top 100 genes by |Spearman’s *ρ*| after filtering). **(B)** Immunoblots showing increased p-AKT (Ser473) and p-mTOR (Ser2448) after *OPCML* knockdown under serum starvation (4 h) and EGF 20 ng/ml restimulation (10 min). The PI3K inhibitor LY294002 (10 μM, 1 h pretreatment) reverses these increases. Densitometry (*right*) normalized to β-Actin. *P*-values by two-tailed tests as specified.

Network analyses of the *OPCML*-related genes highlighted PI3K–AKT as the top enriched pathway (GO/KEGG) (**Figure 5A**). Immunoblotting showed that siOPCML + DMSO increased p-AKT (Ser473) and p-mTOR (Ser2448) compared with siNC + DMSO. LY294002 (10 µM, 1 h) reversed these changes: siOPCML + LY294002 was not different from siNC + LY294002 under the same starvation/EGF conditions (**Figure 5B**).

### 3.6 *OPCML* expression correlates with the immune cell infiltration patterns across cancers and within GBM

**Figure 6:**
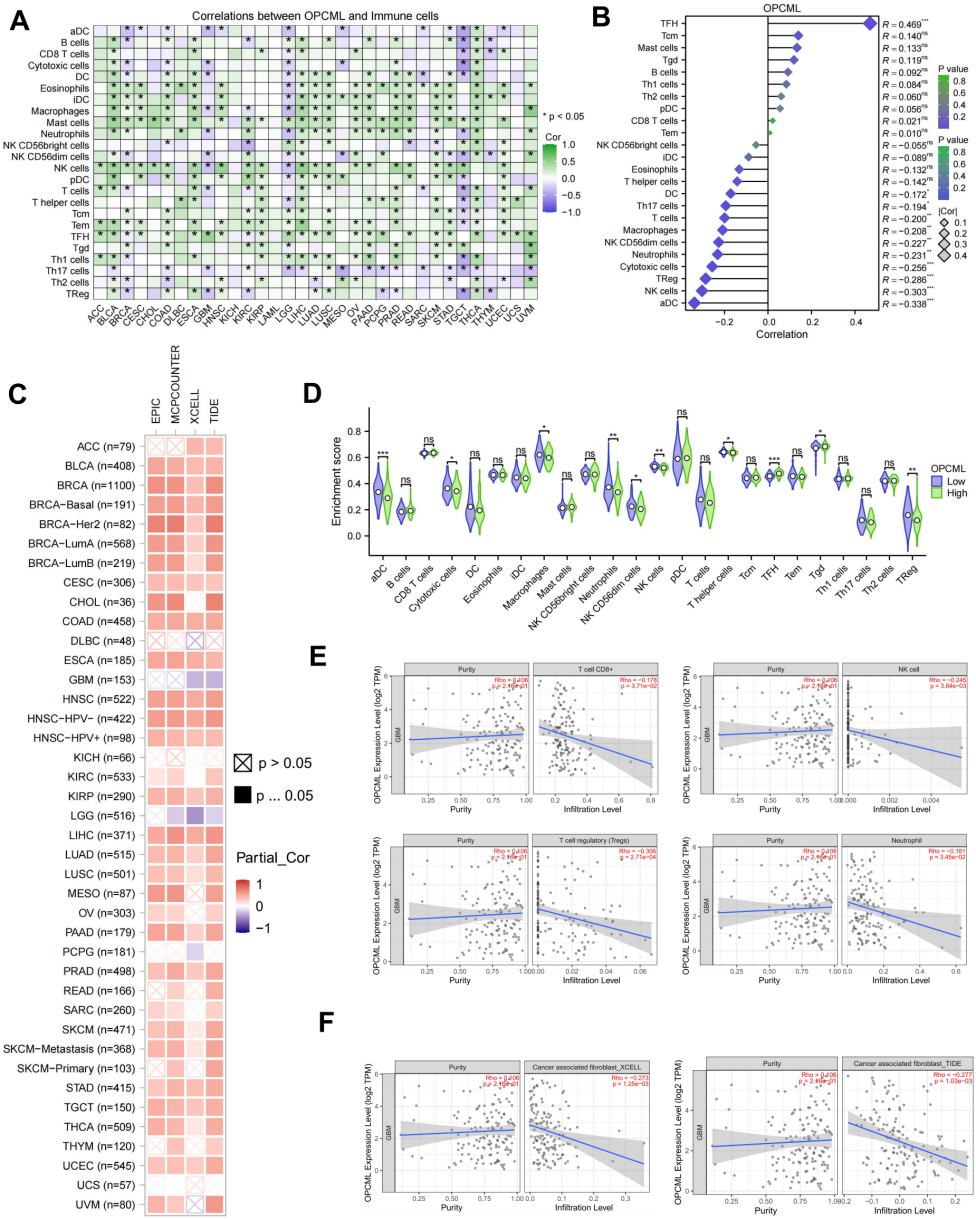
*OPCML* expression and immune cell infiltration patterns. **(A)** Across The Cancer Genome Atlas (TCGA) cancers, *OPCML* generally tracks with higher immune cell signatures [single-sample gene set enrichment analysis (ssGSEA), heat map of Spearman’s correlations]. **(B)** In glioblastoma (GBM), the correlations were reversed for the majority of 24 immune cell types [e.g., natural killer (NK), cytotoxic T, Th17, activated dendritic cells, and neutrophils]. **(C)** TIMER2 estimates show a similar cancer-type switch for CAFs (multiple algorithms). **(D)** GBM high- *vs*. low-*OPCML* groups: the enrichment scores are lower for activated dendritic cells, neutrophils, and NK cells, while the B-cell signatures are largely unchanged (Wilcoxon). **(E)** TIMER2 corroborates the reduced CD8^+^ T cells, regulatory T cells, and neutrophils with higher *OPCML* (sample-level scatter plots with Spearman’s *ρ*). **(F)** Negative association between *OPCML* and cancer-associated fibroblast (CAF) infiltration replicated across two independent algorithms. Multiple testing controlled by Benjamini–Hochberg, where indicated. The asterisk * represents p<0.05, the double asterisk ** represents p<0.001, and the triple asterisk *** represents p<0.0001.

Across the TCGA pan-cancer cohort, the expression of *OPCML* increased in tandem with the majority of the immune cell signatures (**Figure 6A**), suggesting that tumors with higher transcript levels generally harbor denser immune infiltrates. GBM breaks this pattern: for the majority of the 24 ssGSEA cell types—including the Th17, cytotoxic, and natural killer (NK) subsets—the correlations switched to negative (**Figure 6B**). A TIMER2 survey based on four deconvolution algorithms revealed a similar shift for CAFs: while *OPCML* tracked positively with CAF abundance in many cancers, it showed the opposite trend in GBM (**Figure 6C**). When the GBM samples were stratified by gene expression, the high-*OPCML* group displayed significantly lower enrichment scores for the activated dendritic cells, neutrophils, and NK cells, whereas the B-cell signatures remained largely unchanged (**Figure 6D**). TIMER2 corroborated these findings, indicating that an elevated *OPCML* coincides with reduced estimates of CD8^+^ T cells, regulatory T cells, and neutrophils. The sample-level scatter plots confirmed the inverse relationships for these effector populations (**Figure 6E**) and showed a matching negative association with CAF infiltration as assessed using two independent algorithms (**Figure 6F**).

## 4 Discussion

Our data point to a coherent role of *OPCML* in GBM: it is broadly reduced at the bulk level, confined to a small neuron-like compartment at single-cell resolution, and—when silenced—drives a more aggressive behavior via the PI3K–AKT–mTOR axis. *In vitro*, the loss of *OPCML* increased the invasion, migration, and colony growth, with higher p-AKT/p-mTOR that were normalized by PI3K inhibition. Our decision to center the functional work on PI3K–AKT–mTOR was guided by two observations. Firstly, in GBM cohorts, the genes co-varying with *OPCML* were enriched for signaling programs that converge on PI3K–AKT. Secondly, prior epithelial cancer studies have shown *OPCML* to limit surface RTKs (including HER2/EGFR and AXL) and, in doing so, attenuates PI3K–AKT signaling and improves the responses to RTK-directed therapy (11). These converging lines of evidence provided a basis for a potential mechanistic hypothesis that requires further validation in GBM.

Historically, *OPCML* has been reported as downregulated in brain tumors, including GBM, and as epigenetically silenced across multiple cancer types (8, 12). Mechanistic work in epithelial cancers has shown that *OPCML* can act as a cell surface “repressor–adaptor”: by engaging and modulating RTKs, it blunts the downstream signaling and tumor motility (9). One specific example is the *OPCML*–PTPRG axis, which dampens AXL activity and curbs oncogenic signaling (11). In GBM cells, *OPCML* loss elevated p-AKT/p-mTOR, and PI3K blockade reversed these readouts, suggesting that *OPCML* loss may contribute to the activation of this pathway, which is already altered by EGFR amplification/mutation and PTEN loss (13). What is new here is the GBM-specific link: we show that *OPCML* loss is associated with elevated p-AKT/p-mTOR in glioma cells and that PI3K blockade normalizes these readouts, potentially linking a neural adhesion molecule with a canonical survival pathway in this disease context.

The single-cell analysis helped explain why *OPCML* looks low in bulk data, yet remains biologically relevant. In our reanalysis, *OPCML* was largely restricted to a neuron–glia hybrid niche and was scarcely detected in classical malignant clusters. This pattern is in line with IgLON biology—these molecules support neural adhesion, synaptogenesis, and circuit maturation—and it matches our correlation signatures in GBM: genes positively tracking with *OPCML* were enriched for synaptic and ECM organization, whereas negatively tracking genes clustered in cell cycle and replication pathways (6, 14, 15). Put simply, *OPCML* marks a more differentiated, neuron-leaning state. Its loss accompanies a shift toward proliferative and invasive programs.

Clinically, a higher *OPCML* aligned with longer overall survival and retained significance in multivariable models in our analysis. Because the *IDH* status is a key disease determinant, we examined *OPCML* in that context: *OPCML* was generally lower in the *IDH*-wild-type GBM than in the *IDH*-mutant gliomas. This echoes prior work showing that restoring the function of *OPCML* can suppress growth and, in some contexts, sensitize tumors to RTK-directed drugs by disrupting the heterodimerization of HER2–EGFR (10). Structural studies also support cancer-relevant mutations and provide a framework for protein-based restoration strategies (16). While these precedents come from non-glial models, they make a testable case in GBM: *OPCML* restoration (genetic or recombinant) could be paired with inhibitors targeting the EGFR/MEK/ERK and PI3K/AKT/mTOR branches, a combination strategy that has shown superiority over single-pathway blockade in GBM models (17, 18).

The immune infiltration results were deliberately read alongside the unique ecology of GBM. Across cancers, a higher *OPCML* tended to coincide with stronger immune signatures. In GBM, the correlations switched to negative for the majority of cell types (including NK, cytotoxic T, activated dendritic cells, and neutrophils), with parallel trends for the CAF estimates across algorithms. The immune microenvironment of GBM is distinct: myeloid cells (microglia and macrophages) dominate, effective T-cell entry and function are limited, and multiple layers of suppression are common (19, 20). Single-cell and spatial studies have consistently shown that the microglia and infiltrating macrophages are regionally patterned and shape T-cell dysfunction. “More immune” does not necessarily mean “more antitumor” in this disease (21, 22). In that light, the simpler interpretation is that *OPCML*-high tumors may be less inflamed and necrotic, while *OPCML*-low tumors—being more proliferative and invasive—generate chemokine/DAMP landscapes that draw in myeloid cells and inflate the bulk immune scores without improving cytotoxic competence. Notably, CAF-like stromal populations, once thought absent from brain tumors, are now documented by scRNA-seq and spatial profiling and have been linked to immune evasion and poor response to checkpoint blockade in GBM and related central nervous system (CNS) tumors (23, 24). The fact that our deconvolution trends were consistent across the TIMER2 modules adds confidence that the *OPCML*–infiltration relationships are not an artifact of a single method (25).

These mechanistic links suggest straightforward next steps. Firstly, assess whether *OPCML* loss stabilizes or redistributes specific RTKs (e.g., EGFR, PDGFRA, and AXL) at the membrane in GBM lines and patient-derived models. *OPCML*–RTK interactions are documented in other cancers: mapping them in GBM would connect the surface biochemistry to the AKT/mTOR readouts observed here (11). Secondly, as IgLONs anchor adhesion programs, examine the integrin/FAK inputs as a parallel route to PI3K–AKT activation when *OPCML* is depleted—our correlation enrichments for ECM terms make that a plausible branch. Thirdly, extend the pharmacology beyond LY294002 using orthogonal PI3K or AKT inhibitors (e.g., GDC-0084 and MK-2206) and adding *OPCML* re-expression rescue to complete the loss-and-gain logic. This aligns with prior evidence that co-targeting the EGFR/MEK/ERK and PI3K/AKT/mTOR axes outperforms single-node inhibition in GBM models (17). On the immunology side, secretome profiling after *OPCML* perturbation could identify the cytokines and chemokines that link tumor-intrinsic state changes to myeloid recruitment, followed by validation in spatial datasets where regional myeloid/T-cell states are resolved (21).

Several limitations remain. We relied on two immortalized GBM lines (U87 and U251) rather than primary patient-derived cultures or 3D spheroids. As such, the intratumoral heterogeneity and stem-like states are underrepresented. We did not include experiments in primary human GBM cultures in the current study. These models better capture clinical heterogeneity and will be prioritized in subsequent work. We also did not implement an *OPCML* overexpression or re-expression system here. Gain-of-function testing is needed to complement the knockdown data and assess whether an elevated *OPCML* can shift invasive phenotypes. We used one siRNA and two GBM lines. Extending to multiple sequences, gain-of-function rescue, and additional patient-derived models is important. The signaling arm focused on p-AKT and p-mTOR. Including upstream RTK phosphorylation and parallel ERK readouts would refine the pathway map. Our immune analyses relied on deconvolution and enrichment scoring of bulk data. Validation in single-cell or spatial cohorts would better link the *OPCML* levels to specific immune populations *in situ*. Finally, while we saw consistent survival associations, clinical deployment will require prospective testing and integration with established markers (e.g., *IDH* status and methylation classifiers).

In summary, *OPCML* sits at the intersection of neural adhesion programs and canonical oncogenic signaling in GBM. Loss of *OPCML* aligns with proliferative transcriptomic programs, heightens the AKT–mTOR activity, strengthens the invasive and migratory behavior *in vitro*, and associates with a GBM-specific pattern of immune infiltration. In a disease dominated by RTK/PI3K alterations and stubborn resistance to monotherapies, restoring or mimicking *OPCML* function—potentially combined with rational RTK/PI3K axis inhibitors and informed by immune context—deserves further study (26).

## 5 Conclusion

*OPCML* is reduced in GBM and, at single-cell resolution, localizes to a small neuron-leaning compartment. Its loss in U87 and U251 cells increases the invasion, migration, clonogenic growth, and CCK-8 readouts and elevates p-AKT and p-mTOR. These signaling changes are reversed by PI3K inhibition with LY294002. A higher *OPCML* expression aligns with longer survival and a GBM-specific pattern of immune infiltration, linking a neural adhesion program to PI3K–AKT–mTOR activity and microenvironmental tone. Taken together, *OPCML* emerges as both a prognostic marker and a tractable surface modulator that could be leveraged alongside RTK/PI3K axis inhibitors in GBM. Further work should include *OPCML* re-expression rescue and mapping of *OPCML*–RTK interactions in patient-derived models.

## Data Availability

The original contributions presented in the study are included in the article/**Supplementary Material**. Further inquiries can be directed to the corresponding authors.
