# Supplementary material for: Downregulation of OPCML is associated with activation of AKT signaling and aggressive phenotypes in glioblastoma cells
Source: Front Oncol. 2026 Jan 5;15:1710073. doi: 10.3389/fonc.2025.1710073 (PMC12812564; doi:10.3389/fonc.2025.1710073)

Supplement table 1

| **Characteristics** | **Low expression of OPCML** | **High expression of OPCML** | **P value** |
| --- | --- | --- | --- |
| **n** | 84 | 84 |  |
| **Age, n (%)** |  |  | < 0.001 |
| **<= 60** | 31 (18.5%) | 56 (33.3%) |  |
| **> 60** | 53 (31.5%) | 28 (16.7%) |  |
| **Gender, n (%)** |  |  | 0.872 |
| **Female** | 30 (17.9%) | 29 (17.3%) |  |
| **Male** | 54 (32.1%) | 55 (32.7%) |  |
| **Race, n (%)** |  |  | 0.853 |
| **Asian** | 2 (1.2%) | 3 (1.8%) |  |
| **Black or African American** | 5 (3%) | 6 (3.6%) |  |
| **White** | 76 (45.8%) | 74 (44.6%) |  |
| **IDH status, n (%)** |  |  | < 0.001 |
| **WT** | 80 (49.7%) | 69 (42.9%) |  |
| **Mut** | 0 (0%) | 12 (7.5%) |  |
| **Karnofsky performance score, n (%)** |  |  | 0.218 |
| **< 80** | 16 (12.5%) | 20 (15.6%) |  |
| **> 80** | 52 (40.6%) | 40 (31.2%) |  |
| **OS event, n (%)** |  |  | 0.238 |
| **Alive** | 13 (7.7%) | 19 (11.3%) |  |
| **Dead** | 71 (42.3%) | 65 (38.7%) |  |
| **DSS event, n (%)** |  |  | 0.262 |
| **No** | 14 (9%) | 20 (12.9%) |  |
| **Yes** | 63 (40.6%) | 58 (37.4%) |  |
| **PFI event, n (%)** |  |  | 0.238 |
| **No** | 13 (7.7%) | 19 (11.3%) |  |
| **Yes** | 71 (42.3%) | 65 (38.7%) |  |

Supplement Figure 1


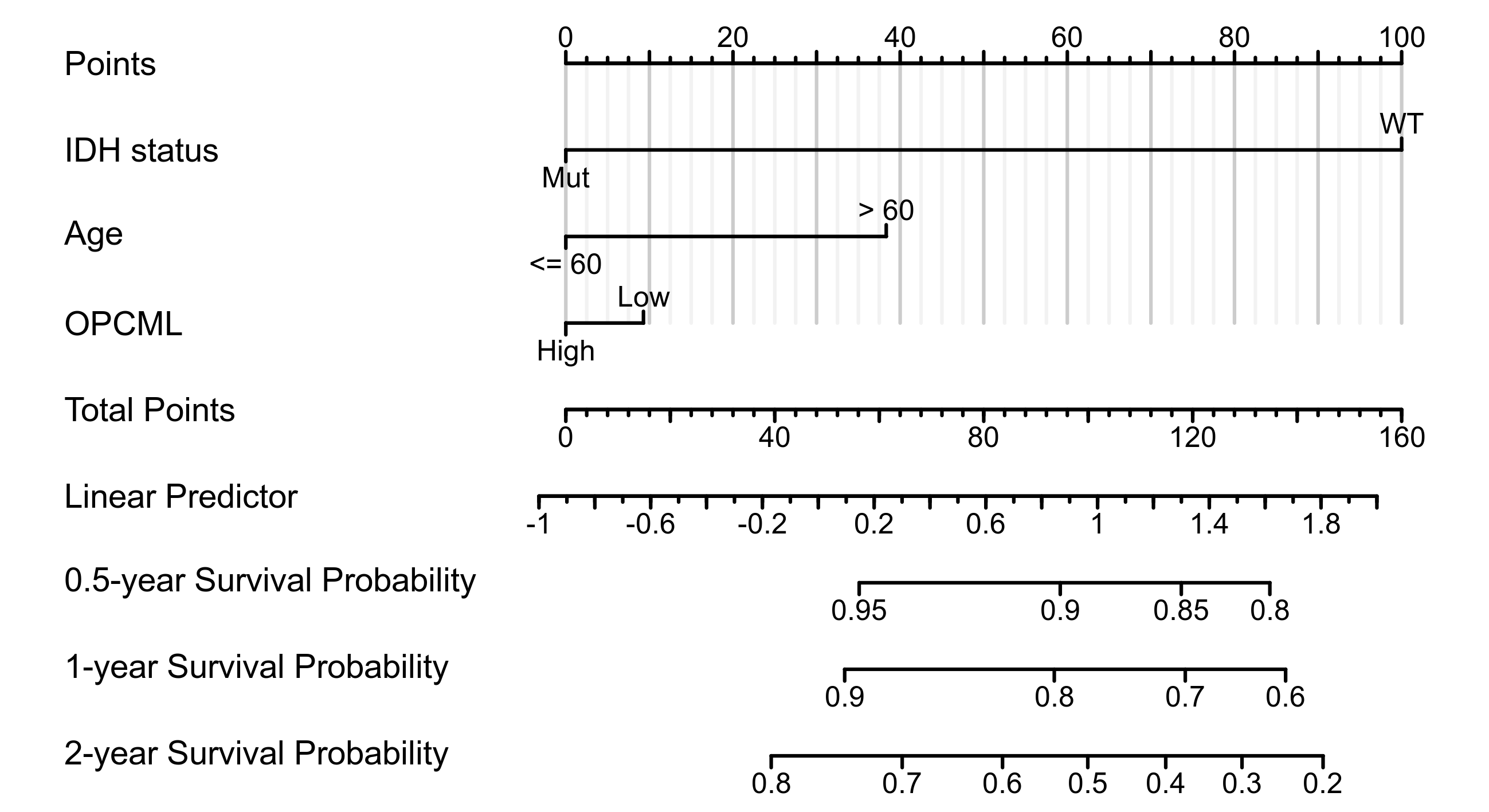


Supplement Figure 2


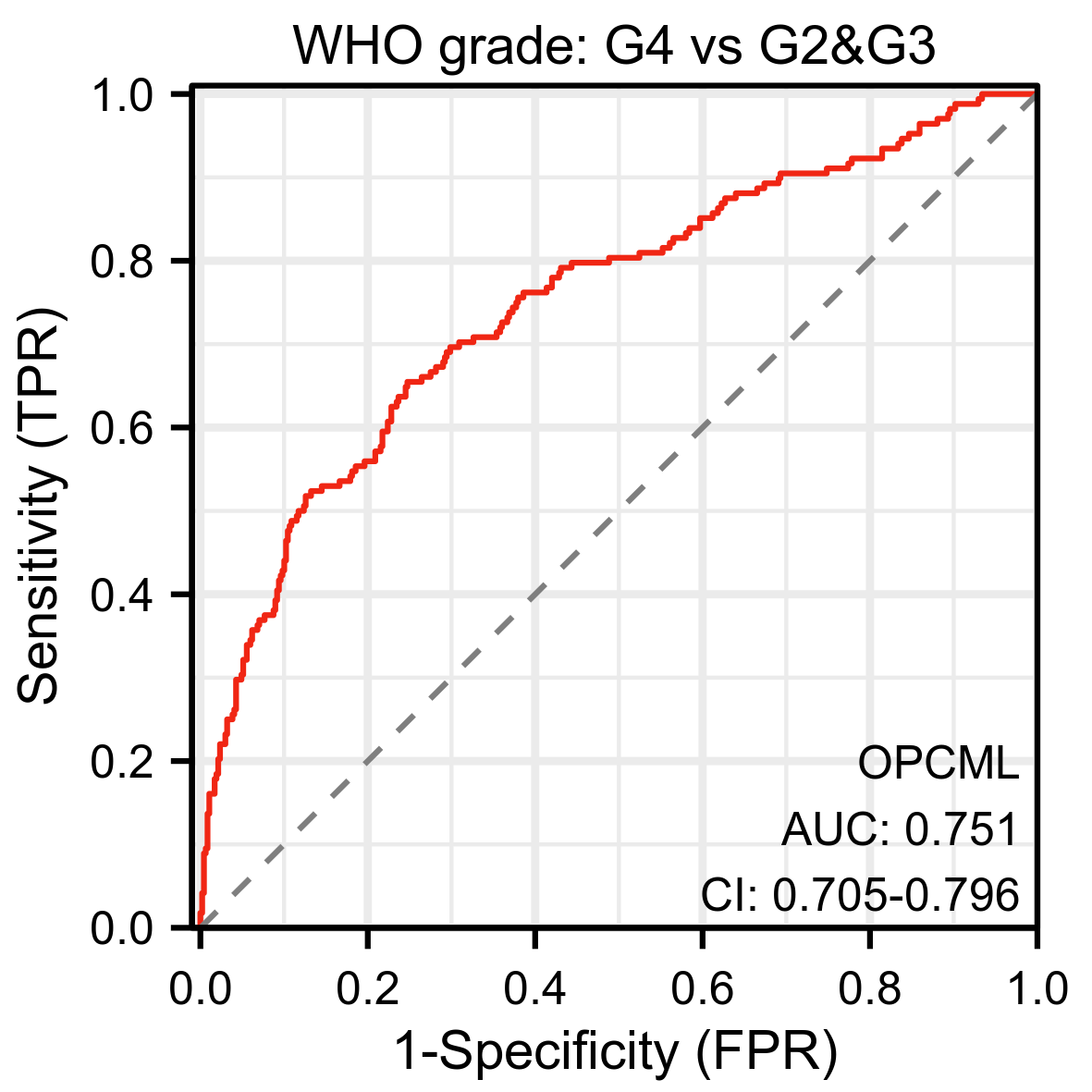

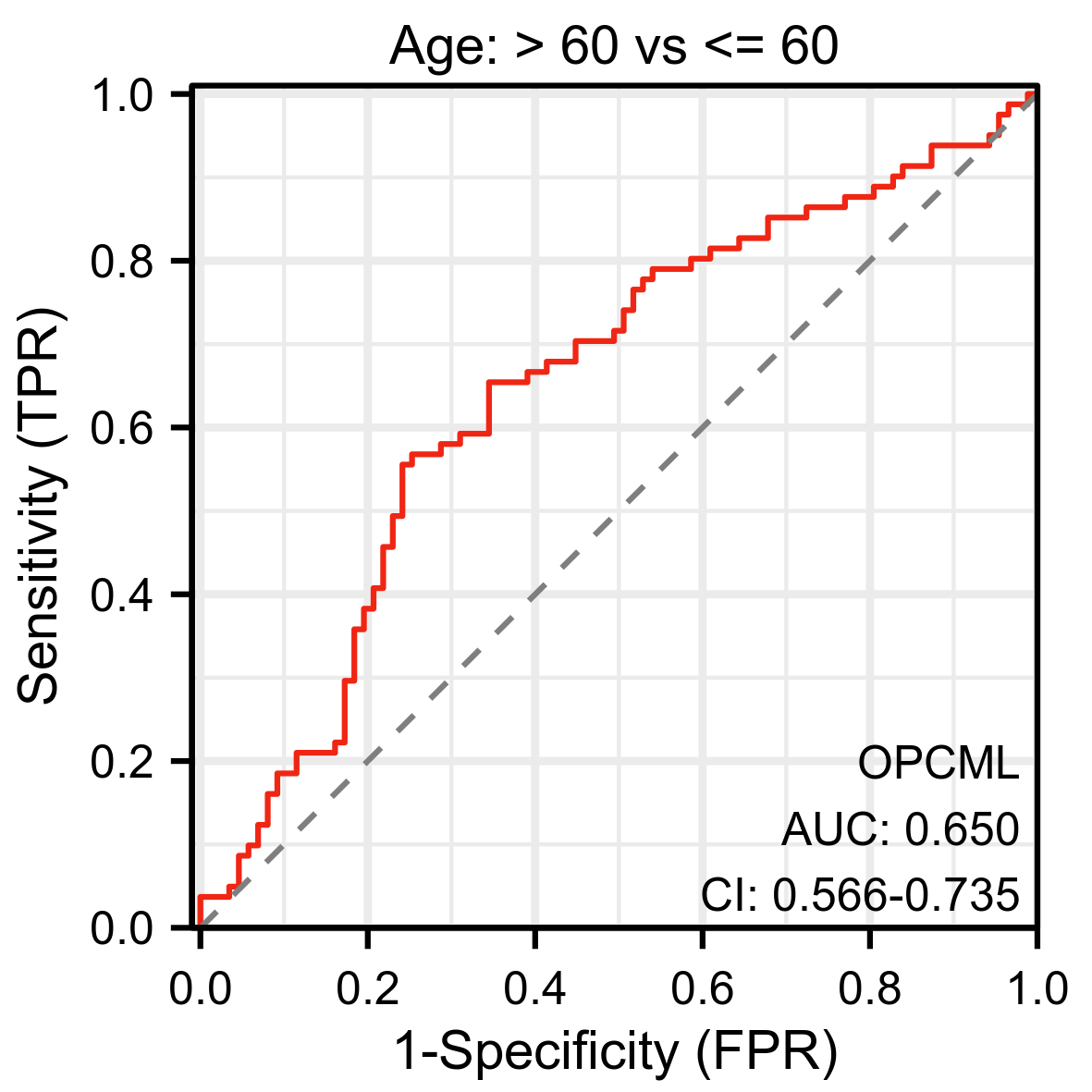

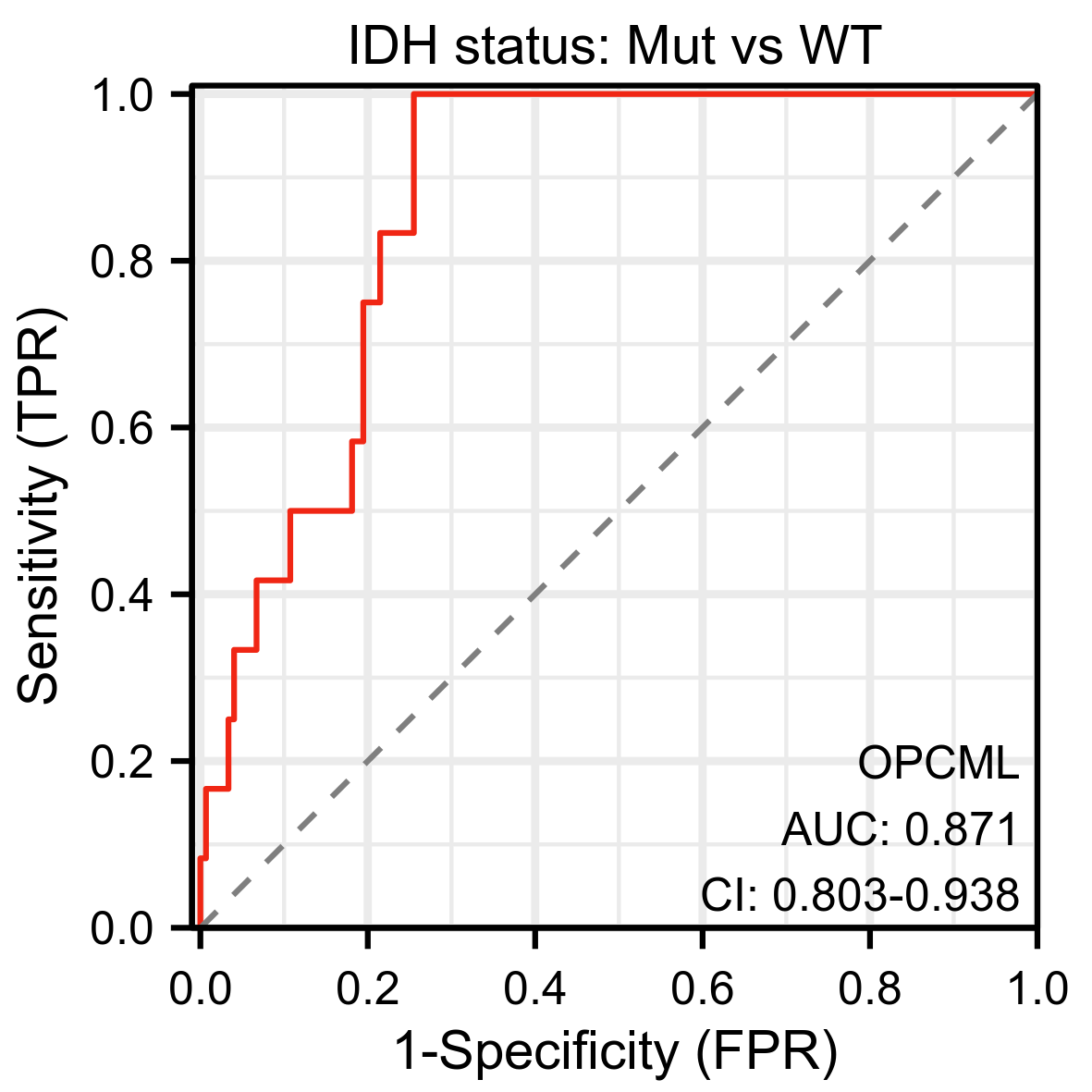

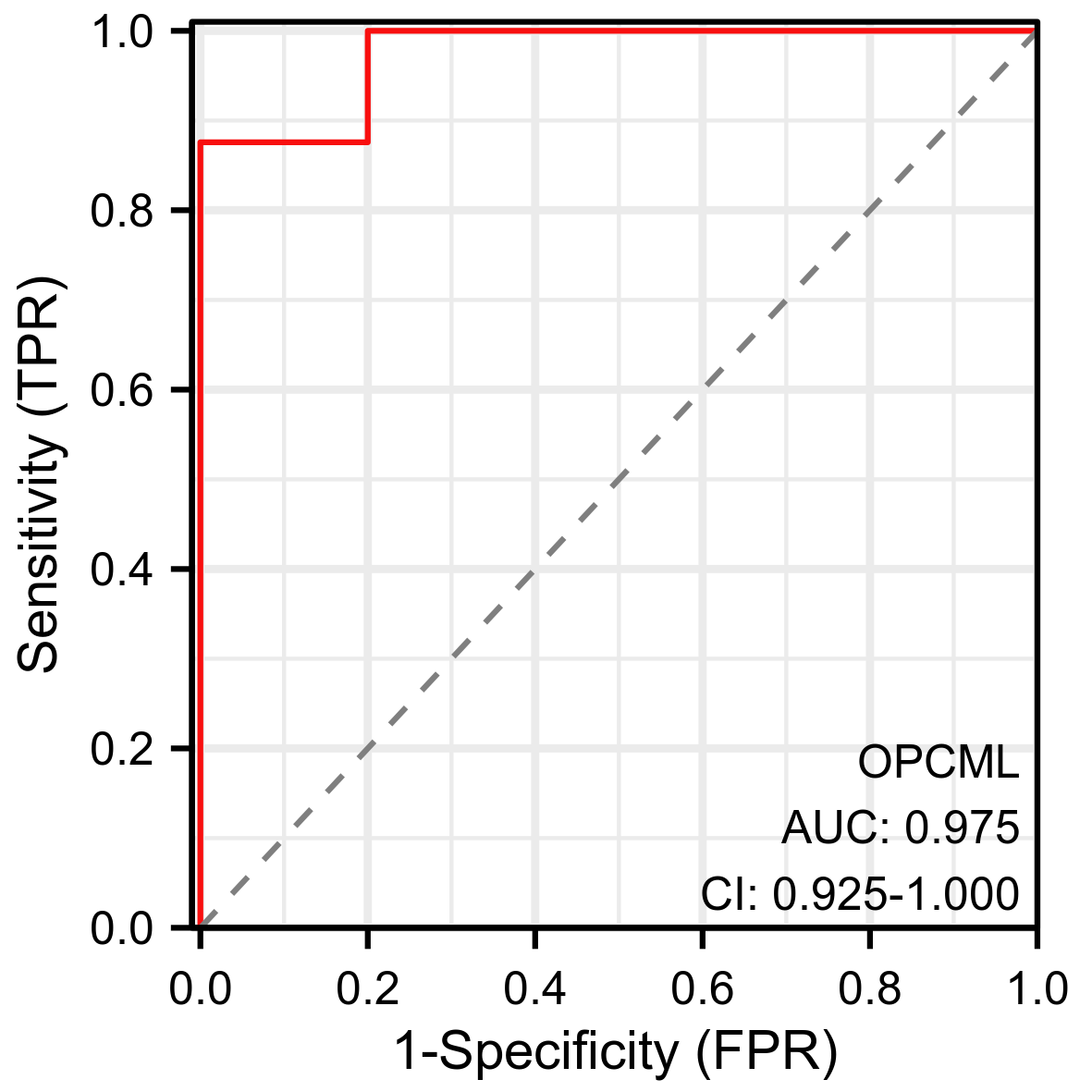


Supplement Figure 3


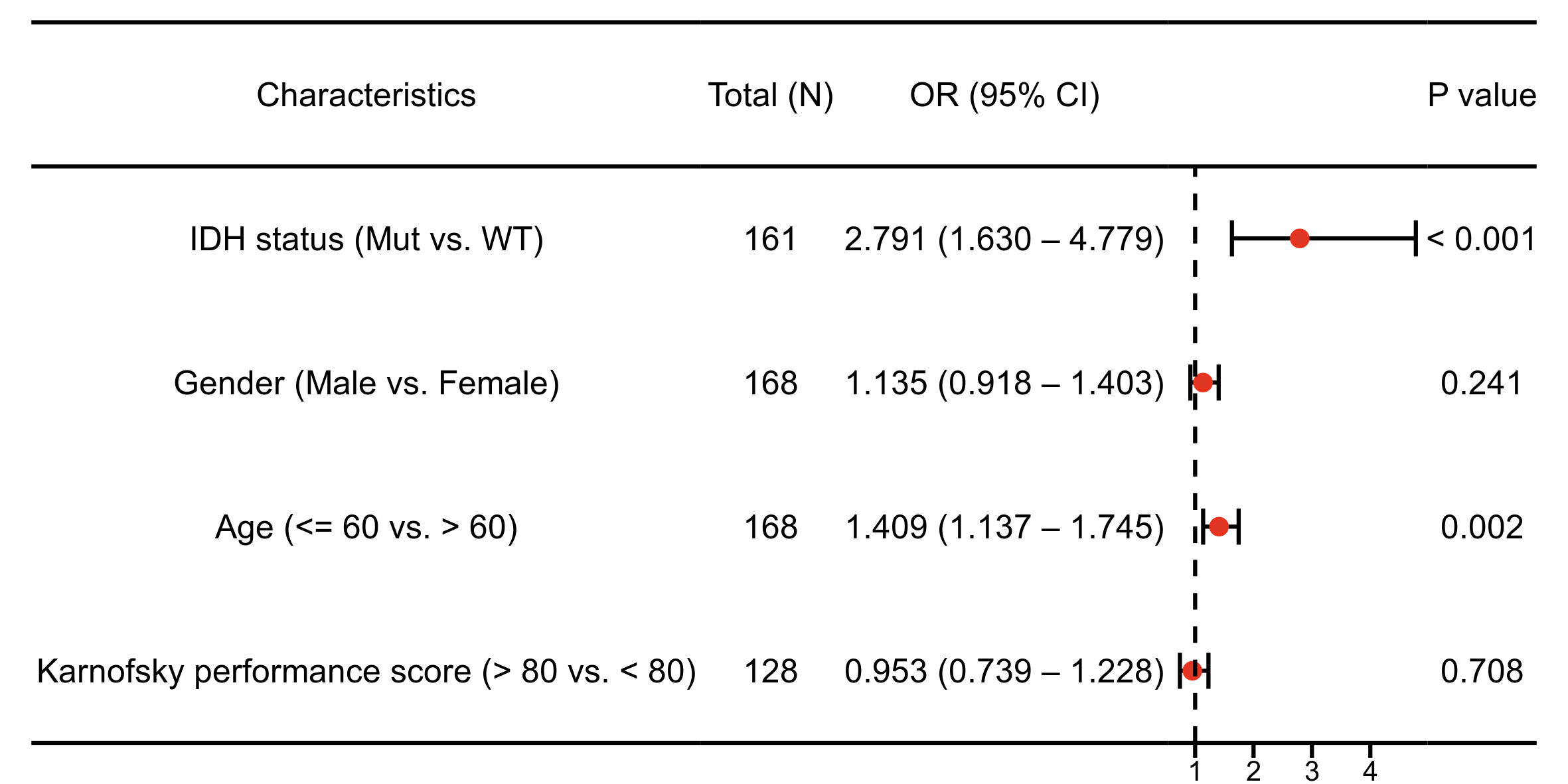

Supplement: Supplementary file 1 [file DataSheet1.docx]
